# Supplementary material for: “The Best Laid Plans”: Do Individual Differences in Planfulness Moderate Effects of Implementation Intention Interventions?
Source: Behav Sci (Basel). 2022 Feb 14;12(2):47. doi: 10.3390/bs12020047 (PMC8869571; doi:10.3390/bs12020047)
Supplement: Supplementary file 1 [file behavsci-12-00047-s001.zip › behavsci-1506316-supplementary.pdf]

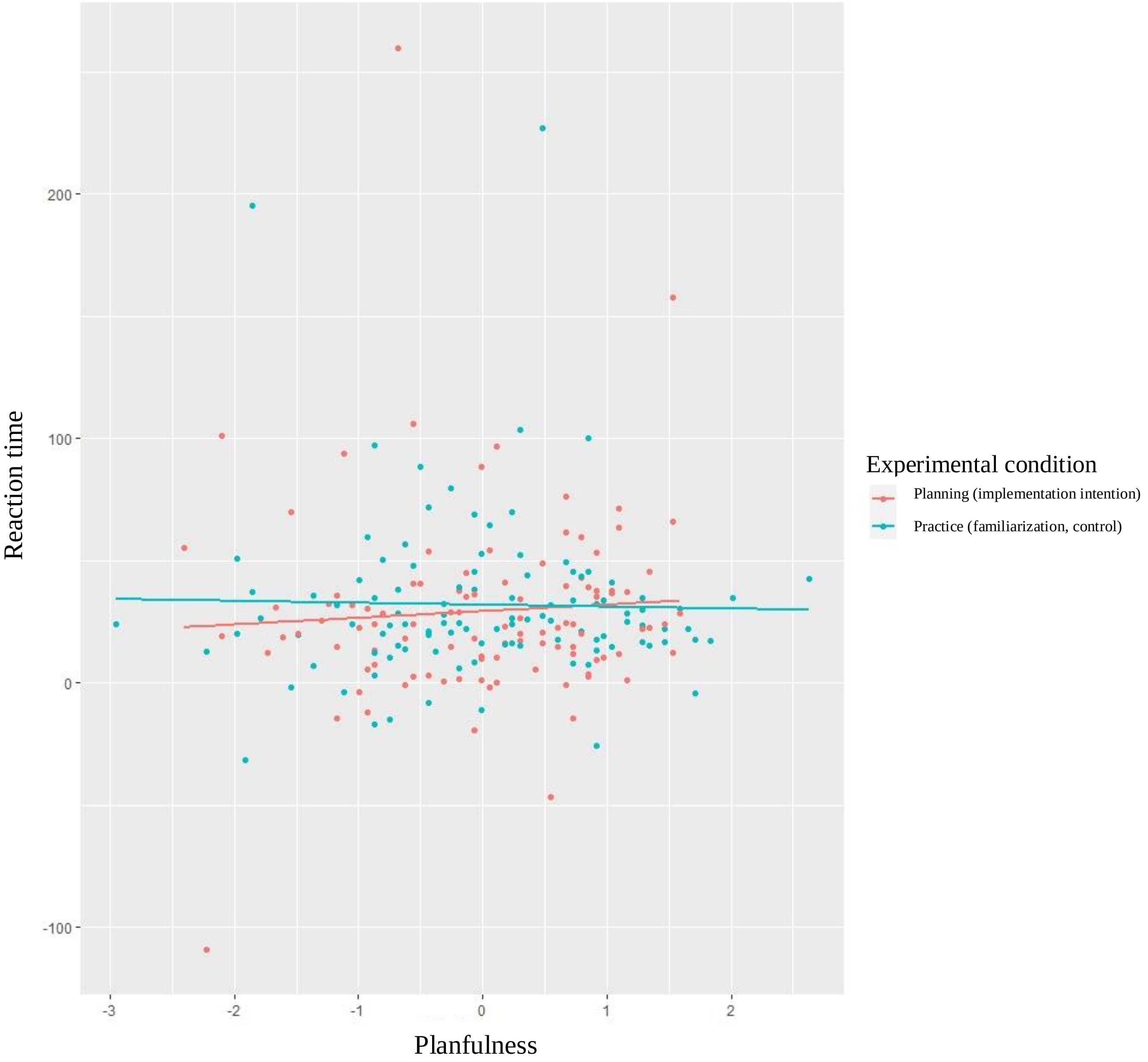

Figure S1. Graph illustrating simple slopes for the effect of planfulness on go no-go task reaction time (RT) for each of the planning (implementation intention) and practice (familiarization, control) experimental conditions.
